# Semi-Supervised Learned Autoencoder for Classification of Events in Distributed Fibre Acoustic Sensors

**DOI:** 10.3390/s25123730

**Published:** 2025-06-14

**Authors:** Artem Kozmin, Oleg Kalashev, Alexey Chernenko, Alexey Redyuk

**Affiliations:** 1 The Artificial Intelligence Research Center, Novosibirsk State University, Pirogova 1, Novosibirsk 630090, Russia; 2 T8 LLC, Krasnobogatyrskaya 44/1, Moscow 107076, Russia

**Keywords:** distributed acoustic sensor, perimeter security system, machine learning, autoencoder, classification, semi-supervised learning, semi-supervised learned autoencoder

## Abstract

The global market for infrastructure security systems based on distributed acoustic sensors is rapidly expanding, driven by the need for timely detection and prevention of potential threats. However, deploying these systems is challenging due to the high costs associated with dataset creation. Additionally, advanced signal processing algorithms are necessary for accurately determining the location and nature of detected events. In this paper, we present an enhanced approach based on semi-supervised learning for developing event classification models tailored for real-time and continuous perimeter monitoring of infrastructure facilities. The proposed method leverages a hybrid architecture combining an autoencoder and a classifier to enhance the accuracy and efficiency of event classification. The autoencoder extracts essential features from raw data using unlabeled data, improving the model’s ability to learn meaningful representations. The classifier, trained on labeled data, recognizes and classifies specific events based on these features. The integrated loss function incorporates elements from both the autoencoder and the classifier, guiding the autoencoder to extract features relevant for accurate event classification. Validation using real-world datasets demonstrates that the proposed method achieves recognition performance comparable to the baseline model, while requiring less labeled data and employing a simpler architecture. These results offer practical insights for reducing deployment costs, enhancing system performance, and increasing throughput for new deployments.

## 1. Introduction

The industry of infrastructure security systems that use distributed acoustic sensors (DAS) is experiencing rapid growth, emerging as a significant and rapidly expanding sector. These systems have a wide range of applications due to their ability to provide continuous real-time monitoring in vast areas. The key segments where DAS are particularly useful include critical infrastructure monitoring (power plants, airports, military bases) [1,2], transportation infrastructure (railways, roads, tunnels) [3,4], pipeline monitoring [5,6,7], building and structural health [8,9], urban public spaces (parks, stadiums, large venues) [10], telecommunication networks, and industrial facilities [11,12]. The primary objective of these systems is not solely the physical protection of facilities, but rather the prompt detection and prevention of potential adverse events, such as activity near the perimeter security zone or attempts to breach protected areas. However, deploying these systems on real sites is challenging due to the high costs associated with replicating relevant events and acquiring large datasets for classification model training, compounded by the limited availability of labeled data within these datasets. Moreover, the sensitivity of distributed acoustic sensors to environmental changes restricts the applicability of data from operational systems to new sites. In addition, advanced signal processing algorithms are necessary to efficiently process large data streams collected from DAS and accurately identify the location and nature of detected events.

In recent years, machine learning (ML) has shown significant potential in the analysis and interpretation of sensor data [13]. ML algorithms excel at identifying complex patterns, learning from them, and making predictions based on the data. The use of neural networks for various classification tasks has become increasingly widespread and essential. However, training neural networks typically requires labeled data, which can be challenging and costly to obtain in real-world applications. Consequently, leveraging unlabeled data has become an important research focus, as it reduces dependence on labeled data and enhances the model’s generalization ability.

For rapid and accurate processing of large data streams from distributed optical sensors, efficient ML-based algorithms can be employed. In [14] the authors propose a long short-term memory (LSTM) model integrated with data denoising techniques for a multipoint fibre Bragg grating sensor system, enhancing the accuracy of signal measurement. In [15] a pattern recognition method for disturbance signals detected by phase-sensitive optical time domain reflectometry distributed optical fibre sensing systems is presented. This method focuses on local disturbance signal details and uses an adaptive denoising technique based on spectral subtraction. The extracted features are classified using an attention-based LSTM network, achieving a higher classification accuracy of 94.3%. In [4] the authors introduce a deep convolutional neural network (CNN) to inspect the state of rail tracks in high-speed railways using DAS technology. Despite achieving a remarkable recognition accuracy of 98.04%, the system encounters challenges arising from the complex track environment and the limited availability of labeled data. In [6] the authors propose an efficient 1-D CNN network and a support vector machine classifier for vibration recognition in pipeline monitoring using DAS. In [7], a convolutional LSTM network was introduced to identify and classify external intrusion events in the optical fibre sensing system used for pipeline safety. The model achieves an average recognition rate of more than 97% for intrusion events and demonstrates effectiveness in the fast and accurate localization of event signals in complex environments.

One of the effective approaches for noise reduction and raw data compression is the use of an autoencoder architecture. This technique extracts a latent representation of the data, which can subsequently be used for event classification. The primary advantage of this approach is that the autoencoder can be trained in an unsupervised mode, utilizing only unlabeled data. However, this method decouples the data compression task from the classification task, as there is no assurance that the compressed representation includes features suitable for effective classification. A refined approach involves a semi-supervised learning technique, where the autoencoder’s training process is additionally directed by external factors.

Table 1 presents key papers dedicated to the application of autoencoders with semi-supervised learning. As shown in the table, this approach has been explored under various conditions and widely applied in image recognition tasks. It is actively used in biology and medicine [16,17], engineering [18], as well as in digit recognition and other image recognition tasks on standard datasets [19,20,21]. It is worth noting that, despite its acceptance in the scientific community, this approach does not have a consistent name and is referred to differently in each paper. One of the key aspects of these works is the variety of loss functions employed. However, fundamentally, all the papers utilize a similar concept and method for training neural networks. The main idea is to train both the autoencoder and the classifier simultaneously. These studies highlight the superiority of this method over the sequential approach, in which the autoencoder is first trained in an unsupervised manner, followed by supervised training of the classifier on the obtained latent representations.

Several successful applications of similar techniques for sensors have been reported [5,22], demonstrating its potential to improve the performance and reliability of sensor systems. In [5] the authors propose a semi-supervised learning approach, combining a sparse stacked autoencoder for feature extraction from unlabeled data and a fully-connected network for event localization using a small amount of labeled data, resulting in improved performance of pipeline safety early warning systems for real-time monitoring of long-distance energy pipelines. Although the paper presents valuable contributions, there are some areas where further clarification or analysis could enhance the robustness of the findings. Specifically, additional labeled data would strengthen confidence in the results, and a more detailed comparison between the proposed model and architectures that combine encoder training with classifiers could provide deeper insights. Additionally, evaluating the model’s throughput capacity, crucial for real-world application, and analysing prediction errors across different approaches would enrich the discussion.

**Table 1:** Overview of applications of semi-supervised learned autoencoders.

Model Name	Application	Loss Functions	Datasets
Semi-Supervised Autoencoder (SSA) [19]	Image Recognition	Reconstr. loss: $L_{MSE}^{rec}$, Sparse loss: $L_{KL}^{spar}$, Logistic loss: $L_{MLE}^{logreg}$, Regularization: $L_{1,2}^{reg}=\left(L_{1}+L_{2}\right)/2$, Total loss: $\alpha L_{MSE}^{rec}+\left(1-\alpha \right)L_{MLE}^{logreg}+\gamma L_{KL}^{spar}+\lambda L_{1,2}^{reg}$	IMAGE [23] IONOSPHERE [24] ISOLET [25] LIRD [26]
Label and Sparse Regularization Autoencoder (LSRAE) [20]	Image Recognition	Reconstr. loss: $L_{MSE}^{rec}$, Sparse loss: $L_{KL}^{spar}$, Class. loss: $L_{MSE}^{cl}$, Regularization: $L_{2}^{reg}$, Total loss: $\alpha L_{MSE}^{rec}+\lambda L_{2}^{reg}+\beta L_{KL}^{spar}+\gamma L_{MSE}^{cl}$	MNIST [27] USPS [28] ISOLET [25]
Semi-Supervised Autoencoder (SS-AE) [21]	Image Recognition (Digital)	Reconstr. loss: $L_{MSE}^{rec}$, Class. loss: $L_{MSE}^{cl}$, Total loss: $L_{MSE}^{rec}+\lambda \cdot L_{MSE}^{cl}$	MNIST [27] USPS [28] CIFAR 10 [29]
Convolutional Autoencoder based Semi-supervised Network (CAESNet) [16]	Optical endomicroscopy, biomedical imaging modality	Reconstr. loss: $L_{MSE}^{rec}$, Class. loss: $L_{CE}^{cl}$, Total loss: $L_{MSE}^{rec}+L_{CE}^{cl}$	Custom dataset 3255 samples Input size: 256×256
Semi-Supervised Autoencoder (SSAE) [17]	Biomedical applications	Reconstr. loss: $L_{Hyber}^{rec}$, Class. loss: $L_{CE}^{cl}$, Total loss: $L_{CE}^{cl}+\lambda L_{Hyber}^{rec}$	Syntetic datasets IPF [30] LUNG [31]
Hybrid Classification Autoencoder (HCAE) [18]	Fault diagnosis in rotating machinery	Reconstr. loss: $L_{BC}^{rec}$, Class. loss: $L_{CE}^{cl}$, Total loss: $L_{BC}^{rec}+\lambda \cdot L_{CE}^{cl}$	Custom dataset 32,722 samples Input size: 64×64

In [22] the authors introduce a semi-supervised framework to enhance the event classification performance of phase-sensitive optical time domain reflectometry using a multitask learning approach. Using a large number of unlabeled samples along with a small number of labeled samples, the proposed model achieves a classification accuracy of up to 96.9%, surpassing other state-of-the-art models. The framework integrates a model that considers both temporal and spatial features, incorporating channel attention and self-attention mechanisms for optimized feature extraction.

In this paper, we introduce a hybrid architecture that combines a semi-supervised learned autoencoder with a classifier, with the aim of improving the accuracy and efficiency of event classification for infrastructure security systems employing distributed acoustic sensors. Our work presents a novel application of this autoencoder training method, utilizing a large dataset comprising more than 380,000 real images obtained under field conditions. The richness and diversity of our dataset allow for highly reliable conclusions about the neural network training process. Unlike previous studies listed in Table 1, our approach employs a simple architecture with a small number of parameters, which facilitates easier training and more reliable comparisons of different approaches. This simplicity also ensures high throughput, which is critically important for real-world deployment of neural networks in perimeter control tasks.

Another important distinction of our work lies in the characteristics of our dataset. Unlike the datasets considered in Table 1, processing of distributed acoustic sensor data involves a significant amount of noise sources and, consequently, a low signal-to-noise ratio [32,33,34,35]. This imposes substantial limitations on the ability to extract important features when using autoencoders. Instead of reconstructing the signal, the autoencoder tends to focus on high-amplitude noise, leading to low-accuracy signal reconstruction. We emphasize that the application of this approach to highly noisy data has not been extensively explored. Additionally, our real data, obtained from various perimeter control stations, presents challenges such as non-stationarity of noise and the dependence of signal amplitude on the observation point of the events.

In addition, the low human interpretability of the observed events in the images is another distinguishing feature of our work. Unlike digit images in datasets such as MNIST [27], events in our dataset are challenging to interpret, complicating the extraction of important features for the classification of violations. Additionally, real-world conditions often involve overlapping events, such as a person’s step and a car’s movement, further complicating classification. For perimeter control tasks, accurately classifying a person’s step amidst more intense events is critical, as misclassification can lead to serious consequences.

Finally, to unify the terminology, we propose introducing a general name for these architectures: semi-supervised learned autoencoder (SSLAE). This name emphasizes the common principle of autoencoder training in this approach. It is important to avoid simpler names, such as semi-supervised autoencoder (SS-AE or SSAE), which can lead to misinterpretation in the scientific community due to the existence of other established abbreviations, such as sparse stacked autoencoder (SSAE).

The main contributions of this study are as follows: (i) the loss function of proposed architecture integrates components from both the autoencoder and the classifier, leveraging the quality of classification to guide the autoencoder’s training and forcing it to extract features that are particularly relevant for accurate event classification; (ii) we assess the accuracy of the SSLAE across varying amounts of labeled data and contrast its performance with a fully-trained classifier under identical conditions, demonstrating the superior performance of SSLAE; (iii) we evaluate the throughput capacity of the SSLAE and compare it with recognized benchmark models such as ResNet and EfficientNet, demonstrating the superior throughput capacity of the SSLAE.

The remainder of the paper is organized as follows. In Section 2, we formulate the problem and provide a brief overview of the considered autoencoder-based approaches. In Section 3, we introduce an event classification approach based on a semi-supervised learned autoencoder. In Section 4, we detail an event classification approach based on autoencoder, where the pre-trained encoder compresses the data before classification. In Section 5, we describe an approach in which the encoder and the classifier are trained jointly, without preliminary autoencoder learning. In Section 6, we compare approaches, present the results, and discuss the model metrics. In Section 7, we evaluate the throughput of the neural network and explain the limitations of the algorithm in the inference mode. In Section 8, we summarize our findings and outline the implications of this research.

## 2. Problem and Dataset Description

### 2.1. Problem Statement and Proposed Solution

DAS uses standard optical fibre as both a transmission medium for light and a sensor to detect acoustic signals and vibrations along its length. The interrogator emits short pulses of laser light into the optical fibre at regular intervals, and as the light travels through the fibre, a small portion scatters back towards the source due to Rayleigh scattering from imperfections in the fibre. A coherent detector measures the phase and amplitude of this backscattered light. Acoustic disturbances or vibrations induce micro-strains, altering the interference pattern, which is detected as changes in phase and amplitude. The signal processing unit analyses these changes to locate and characterize acoustic events, using the time delay between pulse transmission and backscattered light reception to determine the disturbance’s position.

Implementing DAS-based security systems involves processing received signals to accurately classify detected events. These signals are often noisy and contain redundant information, which makes it crucial to extract informative features and eliminate noise to improve classification accuracy. Traditional algorithms like principal component analysis, singular value decomposition, and independent component analysis are commonly used for feature extraction. However, these methods rely on strict assumptions about the data structure, which are often not satisfied in real-world scenarios. A promising alternative for dimensionality reduction and feature discovery is the use of specialized neural network architectures, such as autoencoders. Autoencoders can leverage unlabeled data and enable unsupervised learning through backpropagation, offering a more flexible and effective solution for complex, real-world datasets.

**Figure 1:**
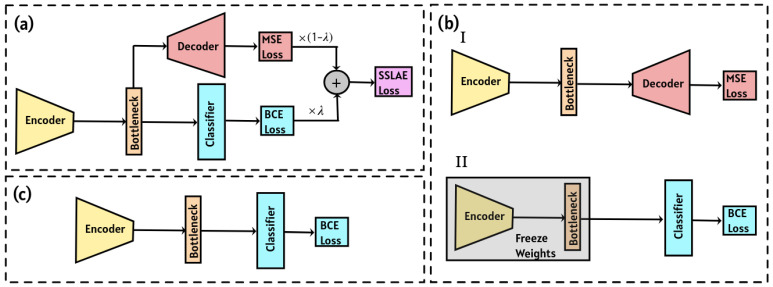
Autoencoder-based schemes for event classification: (**a**) semi-supervised learned autoencoder, (**b**) classifier with pre-trained encoder, (**c**) fully-trained classifier.

The approach we propose in our study, shown in Figure 1a, is a hybrid SSLAE approach, where the autoencoder and classifier are trained simultaneously. The autoencoder is trained on unlabeled data, while the classifier is trained on labeled data. The integrated loss function incorporates elements from both the autoencoder and the classifier, guiding the autoencoder to extract relevant features for accurate event classification. This approach ensures that the training process of the autoencoder is influenced by the quality of the final classification, thus aligning feature extraction with the specific requirements of the classification task.

The second approach, depicted in Figure 1b, involves an autoencoder that is initially trained on unlabeled data, followed by training a classifier block using the encoder block with frozen weights. However, this method disconnects the signal compression stage from the classification stage, as there is no assurance that the compressed representation includes features suitable for effective classification.

Finally, we consider the approach illustrated in Figure 1c, which addresses the aforementioned issue by simultaneously training the encoder and classifier blocks. In this case, the training metric for encoder weights is classification accuracy rather than reconstruction accuracy. However, this approach requires more labeled data due to the increased complexity of the architecture and the larger number of trainable parameters.

### 2.2. Dataset Description

**Figure 2:**
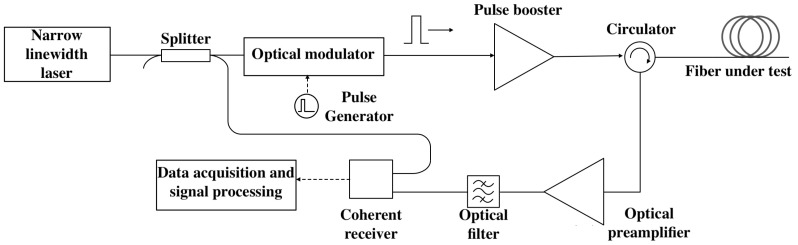
Scheme of the experimental setup.

The dataset consists of images with dimensions of 96 × 64 pixels in three colour channels, collected from seven operational perimeter security systems with the distributed acoustic sensor “Dunay”, developed by T8 LLC. A scheme of the experimental setup is shown in Figure 2 and is described in detail in [36], which outlines the signal acquisition system and phase-based analysis of dynamic strain along the optical fibre. In the DAS system, probe pulses are generated from the continuous-wave output of a narrow-linewidth, single-frequency laser operating at a wavelength of 1550 nm using an optical modulator. These pulses are then amplified by an EDFA booster. The DAS is connected to a fibre line composed of standard single-mode fibre. The backscattered optical signal is routed through an optical circulator to the input of an EDFA preamplifier for additional amplification. After amplification, the signal is filtered by an optical filter and delivered to a coherent receiver. Subsequent signal processing is carried out by a digital signal processing unit.

The raw signal from the device is a set of waveforms recorded at a sampling rate of 1 kHz, with a spatial resolution of 1.6 m. To construct RGB images, these waveforms are filtered using Butterworth bandpass filters in three frequency bands: 4–10 Hz (red channel), 20–40 Hz (green channel), and 35–40 Hz (blue channel), followed by downsampling to 20 Hz. These bands were chosen to maximize the signal-to-noise ratio based on preliminary experiments. The specific selection depends on the type of target events, the prevailing noise conditions, and signal attenuation characteristics, which can vary by location. The 20–40 Hz frequency range is generally well-suited for detecting footsteps. However, in some environments—such as areas near highways—it is often contaminated by background noise. In these cases, a narrower band of 35–40 Hz can still capture footstep signals effectively, particularly when the fibre is not deeply buried. To ensure reliable detection across varying conditions, both frequency bands are used in parallel.

While three filters generally provide sufficient information for robust classification and are convenient for RGB-based visualization, we note that for specific tasks such as human step detection, a reduced set of two channels (green and blue) can be used with minimal impact on performance. The red channel is most relevant for detecting heavier disturbances such as machinery activity. The scale of each resulting image corresponds to a time window of 4.8 s (with lines recorded every 0.05 s) and a spatial extent of 102.4 m (with sensors spaced 1.6 m apart along the optical fibre).

In total, the dataset consists of 299,481 unique images for training, 65,186 for validation, and 19,411 for testing process. Each image is labeled into one of three distinct classes, representing events detected during perimeter control. The most common class, labeled as “noise”, represents events that are not considered significant. This class constitutes 70.6% of the training dataset, 71.5% of the validation dataset, and 69.4% of the test dataset. The next prevalent class, labeled as “human_step”, denotes events associated with human steps, comprising 19.4% of the training, 18.2% of the validation and 19.9% of the test data. The least common class, “human_digg”, denotes digging, making up 10.0% of the training, 10.3% of validation, and 10.7% of the test set. Among the examples in the “noise” class are recordings of passing vehicles, heavy transport activity, engine idling noise, excavation by heavy machinery, and cases where the acoustic event is only partially captured at the edge of the sample.

In our study, we focused on a binary classification task where “human_step” served as the positive class, and we combined “noise” and “human_digg” into the negative class. The proportion of the positive class was 19.4%, 18.2%, and 19.9% in the training, validation, and test datasets, respectively.

## 3. Semi-Supervised Learned Autoencoder for Event Classification

In this section, we introduce the semi-supervised learned autoencoder approach schematically illustrated in Figure 1a. SSLAE is a neural network architecture that combines unsupervised learning (autoencoder) and supervised learning (classifier). It extends the concept of traditional autoencoders by incorporating labeled data into the training process, allowing the model to learn from both the reconstruction task and the labeled information.

The architecture of SSLAE comprises an encoder (Table A1 of Appendix A. Basic Blocks), a decoder (Table A2), and a classifier (Table A3), as depicted in Figure 1a. The encoder processes input data sized 96 × 64 pixels in three channels, converting it into a lower-dimensional hidden representation of 12 × 8 pixels in three channels. The decoder then attempts to reconstruct the original input from this hidden representation. Similarly to traditional autoencoder training, reconstruction losses are computed by comparing the decoder’s output with the input, with the network trained to minimize these losses using the mean squared error (MSE) loss function. In addition, a binary classifier is incorporated to predict the data labels using the same hidden representation generated by the encoder. Classification losses are computed by comparing the predicted labels with the actual labels in the labeled samples, utilizing the binary cross-entropy (BCE) loss function, which quantifies BCE between the target and predicted probabilities: $BCE=-\left(1/N\right)\sum_{i=1}^{N}y_{i}\log\left(p(y_{i})\right)+\left(1-y_{i}\right)\log\left(1-p(y_{i})\right),$ where $y_{i}$ is the target class (0 for negative and 1 for positive) and $p(y_{i})$ is the predicted probability of observing the positive class, which in our case represents a human step.

The overall objective function is a combination of reconstruction loss and classification loss, defined as $SSLAE_{loss}=\lambda \cdot BCE+\left(1-\lambda \right)\cdot MSE$, where λ is a hyperparameter that balances the priority between classification and image reconstruction tasks. When λ=0, the model trains only the autoencoder using MSE as the loss function, while λ=1 corresponds to the fully-trained classifier case. The model is trained to minimize both losses simultaneously, allowing it to learn compact data representations while effectively utilizing labeled information for supervised tasks. Therefore, in the semi-supervised mode, the encoder is trained to capture both the internal structure of the data for reconstruction and meaningful features for classification.

During each training epoch, the entire training dataset was used to compute both the MSE metric for the autoencoder and the BCE metric for the classifier branch. Subsequently, the weights were updated. The algorithm for supervised training of the SSLAE is shown in Algorithm 1.
**Algorithm 1:** SSLAE algorithm for supervised learningInput: 3-channel image (X) obtained after applying Butterworth filters, label (Y) of this image.
Parameters: The batch size $\left(m_{ae}\right)$ of autoencoder, initial learning rate (α), waiting parameter $\left(\eta_{\alpha }\right)$ without loss improvement, learning rate reduction factor $\left(\beta_{\alpha }\right)$, priority parameter (λ) between solutions to classification and reconstruction problems, autoencoder and classifier iterations per loop $\left(n_{ae}\right)$.
Require:Initial Encoder $E(\;\cdot \;;\theta_{e^{0}})$, Decoder $D(\;\cdot \;;\theta_{d^{0}})$, Classifier $C(\;\cdot \;;\theta_{c^{0}})$  1:while $\theta_{e},\theta_{d},\theta_{c}$ has not converged do  2:     for$\;i\in [1,n_{ae}]$ do  3:          for$\;j\in [1,m_{ae}]$ do  4:               Shuffle and sample the data image x∼X, label y∼Y.  5:               $\hat{f_{e}}\leftarrow E\left(x;\theta_{e}\right)$  6:               $\hat{x},\;\hat{y}\leftarrow D\left(\;\hat{f_{e}}\;;\theta_{d}\right),\;C\left(\;\hat{f_{e}}\;;\theta_{c}\right)$  7:            end for  8:            $L_{c}\leftarrow \sum_{j=1}^{m_{ae}}L_{BCE}\left(y_{j},\;\hat{y_{j}}\right)$  9:            $L_{ae}\leftarrow \sum_{j=1}^{m_{ae}}L_{MSE}\left(x_{j},\;\hat{x_{j}}\right)$10:            $L_{sslae}\leftarrow \lambda \cdot L_{c}+\left(1-\lambda \right)\cdot L_{ae}$11:            $\nabla_{\theta_{e},\theta_{d},\theta_{c}}L_{sslae}\leftarrow \lambda \cdot \nabla_{\theta_{e},\theta_{c}}L_{c}\;+\left(1-\lambda \right)\cdot \nabla_{\theta_{e},\theta_{d}}L_{ae}$12:            $\theta_{e},\theta_{d},\theta_{c}\leftarrow Adam\left(\nabla_{\theta_{e},\theta_{d},\theta_{c}}L_{sslae},\;\alpha \right)$13:    end for14:    $\alpha \leftarrow ReduceLROnPlateau(L_{sslae}^{valid},\alpha ,\eta_{\alpha },\beta_{\alpha })$15:end while


A separate validation dataset, containing 65,186 images, was utilized for hyperparameter optimization. The test dataset included 19,411 images. During training, a learning rate reduction algorithm was employed when the validation error plateaued, ensuring optimal convergence. For hyperparameter tuning, we used the open-source Optuna library with the HyperbandPruner [37] and TPESampler [38] algorithms. The optimal hyperparameters were determined through 150 trials. The Adam optimizer was used as the optimization algorithm. The adjustable hyperparameters included batch size (1024 to 8192), learning rate ($10^{-5}$ to $10^{-1}$), learning rate reduction factor (0.1 to 0.9), and patience parameter (2 to 7 epochs). The hyperparameter λ was also optimized within a range of 0 to 1. The number of training epochs was set at 100. The primary metric used for model selection was the accuracy in the validation set. Furthermore, we employed the area under the ROC curve (AUC ROC) as a secondary metric. We achieved the highest accuracy levels of 98.12%, 96.98%, and 97.01% on the training, validation, and test datasets, respectively. The AUC ROC levels were 0.9969, 0.9909, and 0.9909, respectively.

To evaluate the performance of the autoencoder, the peak signal-to-noise ratio (PSNR) was used as the primary metric, measuring the ratio between the maximum possible signal value and the level of noise distorting the signal: $PSNR=10\log_{10}\left(MAX_{I}^{2}/MSE\left(I,P\right)\right)$, where $MAX_{I}$ represents the maximum intensity of the input image *I*, and MSE(I,P) denotes the mean squared error between the input image *I* and the image *P* produced by the autoencoder. The average PSNR values were 21.46 dB, 21.44 dB, and 21.54 dB for the training, validation, and test datasets, respectively.

The confusion matrices for all datasets are presented in Table 2. It allows to calculate the main metrics used in the classification, such as Precision, Recall, and F1 score using the following formulas:

$$Precision=\frac{TP}{TP+FP},\;Recall=\frac{TP}{TP+FN},\;F1=2\cdot \frac{Precision\cdot Recall}{Precision+Recall},$$

where TP (TruePositive) is the correctly predicted positive result (for the test dataset TP=3465), FP (FalsePositive) is the incorrectly predicted positive result (FP=186), and FN (FalseNegative) is the incorrectly predicted negative result (FN=395).

## 4. Classifier with Pre-Trained Encoder

In this section, we describe an alternative classification approach for comparison with the proposed SSLAE method, as schematically illustrated in Figure 1b. In this method, we train an autoencoder to extract informative features from the data using our dataset. For the classification task, we utilize the encoder from the pre-trained autoencoder with frozen weights and add a classification block that is trained on labeled data.

### 4.1. Autoencoder

We employ an autoencoder architecture consisting of two consecutive blocks: an encoder (detailed in Table A1) and a decoder (detailed in Table A2), as illustrated schematically in Figure 1b. This autoencoder is designed to reduce the dimensionality of the input image by a factor of 64, effectively mapping the original high-dimensional space into a significantly lower-dimensional representation. The autoencoder was trained using a dataset comprising 299,481 unique images, each with dimensions of 96 × 64 pixels and 3 colour channels. The training goal was to reconstruct the original images with high accuracy, evaluated using the MSE loss function. A separate validation dataset, containing 65,186 images, was utilized for hyperparameter optimization. The test dataset included 19,411 images. During training, a learning rate reduction algorithm was employed when the validation error plateaued, ensuring optimal convergence.

The optimal hyperparameters were determined through 60 trials. The key tuned hyperparameters included batch size (128 to 512), learning rate ($10^{-5}$ to $10^{-1}$), learning rate reduction factor (0.1 to 0.9) and patience (2 to 10 epochs). Training was carried out for a total of 100 epochs.

**Figure 3:**
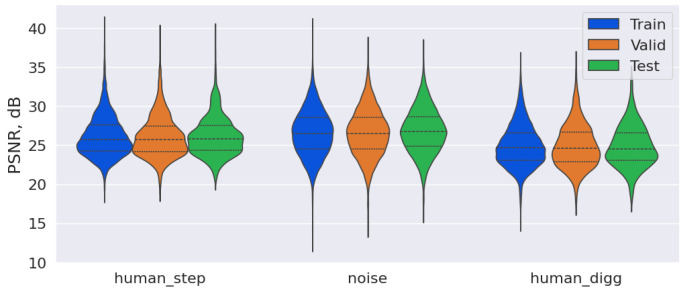
Violin plot of PSNR for images compressed by a factor of 64 using the autoencoder.

The best learned autoencoder model, which compresses images by a factor of 64, achieved average PSNR values of 26.32 dB on the training, 26.30 dB on the validation, and 26.45 dB on the test dataset. Figure 3 presents violin plots of PSNR for different datasets and classes, where the dotted lines indicate the quartile values, and the shape of the curve reflects the kernel density estimate for PSNR.

**Figure 4:**
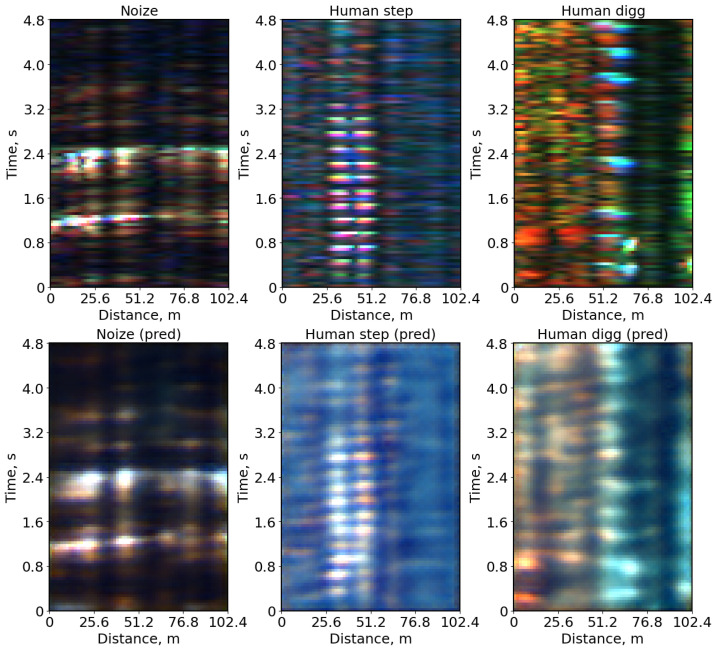
Original (**top row**) and reconstructed (**bottom row**) images from the autoencoder after compression by a factor of 64. The PSNR values from left to right are: “noise”—29.71 dB, “human_step”—26.83 dB, “human_digg”—24.59 dB.

As shown in Figure 3, there is a significant variation in the PSNR values for the class “noise” during compression. This variation can be attributed to the diversity within this class, which includes images with background noise, as well as images of vehicles and other moving objects. Additionally, similar PSNR distributions across the training, validation, and test datasets indicate the absence of overfitting in this model. This conclusion is further supported by a comparison of the MSE errors in these datasets. Figure 4 presents the original and reconstructed images for different classes following compression by a factor of 64, which correspond closely to the original images.

### 4.2. Classifier

To address the binary classification task, we utilize the encoder block from the previously trained autoencoder as the feature extraction module for our classifier. We then integrate a classification block (see Table A3) with this encoder. During the training process, the weights of the encoder layers are kept frozen, enabling only the classifier to be trained.

To train our classifier, we employed the BCE loss function. We used the Adam optimizer with learning rate reduction applied on a plateau. The training, validation and test datasets were the same as those used for autoencoder training. The optimal hyperparameters were determined using the Optuna library with HyperbandPruner and TPESampler. The total number of trials was set to 150. Key tuned hyperparameters included: batch size (1024 to 8192), learning rate ($10^{-5}$ to $10^{-1}$), learning rate reduction factor (0.1 to 0.9) and patience parameter (2 to 7 epochs). The number of training epochs was fixed at 50.

The primary metric used for model selection was the accuracy in the validation set. The highest accuracy results were 87.16%, 87.52%, and 86.95% for the training, validation, and test datasets, respectively. The corresponding AUC ROC scores were 0.9010, 0.8984, and 0.9004. The confusion matrices for all datasets are presented in Table 3.

The results show that while the PSNR level for SSLAE is lower compared to the classifier with a pre-trained encoder (21.54 vs. 26.45 dB for test dataset), the classification accuracy is significantly higher (97.01% vs. 86.95% for test dataset). This reaffirms that SSLAE encourages the encoder to extract features essential for accurate classification.

## 5. Fully-Trained Classifier

In this section, we describe the third classification approach, schematically illustrated in Figure 1c. This method employs a neural network that comprises both an encoder (Table A1) and a classification block (Table A3). Unlike the previous approach, both components were trained simultaneously from random parameter initialization, using the same learning rate and the BCE loss function to evaluate the error. The training, validation and test datasets remained consistent with those used in the previous section.

During the optimal hyperparameter tuning, the total number of trials was set to 150. The key adjusted hyperparameters included batch size (2048 to 8192), learning rate ($10^{-5}$ to $10^{-1}$), learning rate reduction factor (0.1 to 0.9) and patience parameter (2 to 7 epochs). The number of training epochs was fixed at 100. The accuracy in the validation set was the primary metric for model selection. We achieved the highest accuracy levels of 98.02%, 97.16%, and 97.06% on the training, validation, and test datasets, respectively. The AUC ROC levels were 0.9960, 0.9919, and 0.9904. The confusion matrices for all datasets are presented in Table 4.

The results of this approach are significantly superior to those of the classifier with a pre-trained encoder (Figure 1b). This is due to the encoder being trained to enhance classification accuracy, whereas in the second approach the autoencoder is trained to minimize reconstruction error. Consequently, the encoder produces distinct latent representations in each method.

## 6. Comparison of SSLAE and Fully-Trained Classifier

As shown in the previous section, both the fully-trained classifier (FTC) and the SSLAE approach achieve comparable accuracy, using the entire set of labeled training dataset for training. However, a notable advantage of the SSLAE approach is its flexibility in the amount of data used to train the autoencoder and the classifier branch. The classifier can be trained with limited labeled data, which is common in real-world scenarios, while the autoencoder can use the available unlabeled data for training.

In this section, we compare the SSLAE and FTC approaches. To highlight the advantage of SSLAE when using unlabeled data, we split the labeled data from the training and validation sets into partially labeled and conditionally unlabeled data in equal proportions. For example, with 10% labeled data, the training set had 29,948 labeled samples and 269,533 conditionally unlabeled samples, while the validation set had 6518 labeled samples and 58,668 conditionally unlabeled samples. The test set remained unchanged with 19,411 labeled samples.

The FTC was trained only on labeled samples, whereas the SSLAE utilized both labeled and conditionally unlabeled samples. For the SSLAE model, the error on unlabeled data was computed using only the MSE, with the BCE set to zero. To manage this training process, we created a dictionary that maps each sample to a binary flag: True for labeled and False for conditionally unlabeled. This ensured that no data was leaked between labeled and unlabeled samples. The algorithm for training the semi-supervised learned autoencoder is shown in Algorithm 2.
**Algorithm 2:** SSLAE algorithm for supervised learning with partially labeled dataInput: 3-channel image (X) obtained after applying Butterworth filters, label (Y) of this image, flag (Ξ) for use as a labeled sample.
Parameters: The batch size $\left(m_{ae}\right)$ of autoencoder, initial learning rate (α), waiting parameter $\left(\eta_{\alpha }\right)$ without loss improvement, learning rate reduction factor $\left(\beta_{\alpha }\right)$, priority parameter (λ) between solutions to classification and reconstruction problems, autoencoder and classifier iterations per loop $\left(n_{ae}\right)$.
Require:Initial Encoder $E(\;\cdot \;;\theta_{e^{0}})$, Decoder $D(\;\cdot \;;\theta_{d^{0}})$, Classifier $C(\;\cdot \;;\theta_{c^{0}})$     while $\theta_{e},\theta_{d},\theta_{c}$ has not converged do  2:      for $i\in [1,n_{ae}]$ do                 for $j\in [1,m_{ae}]$ do  4:                 Shuffle and sample the data image x∼X, label y∼Y, flag ξ∼Ξ.                       $\hat{f_{e}}\leftarrow E\left(x;\theta_{e}\right)$  6:                 $\hat{x},\;\hat{y}\leftarrow D\left(\;\hat{f_{e}}\;;\theta_{d}\right),\;C\left(\;\hat{f_{e}}\;;\theta_{c}\right)$                end for  8:           $L_{c}\leftarrow \sum_{j=1}^{m_{ae}}L_{BCE}\left(y_{j}\cdot \xi_{j},\;\hat{y_{j}}\cdot \xi_{j}\right)$                $L_{ae}\leftarrow \sum_{j=1}^{m_{ae}}L_{MSE}\left(x_{j},\;\hat{x_{j}}\right)$10:           $L_{sslae}\leftarrow \lambda \cdot L_{c}+\left(1-\lambda \right)\cdot L_{ae}$                $\nabla_{\theta_{e},\theta_{d},\theta_{c}}L_{sslae}\leftarrow \lambda \cdot \nabla_{\theta_{e},\theta_{c}}L_{c}\;+\left(1-\lambda \right)\cdot \nabla_{\theta_{e},\theta_{d}}L_{ae}$12:           $\theta_{e},\theta_{d},\theta_{c}\leftarrow Adam\left(\nabla_{\theta_{e},\theta_{d},\theta_{c}}L_{sslae},\;\alpha \right)$           end for14:      $\alpha \leftarrow ReduceLROnPlateau(L_{sslae}^{valid},\alpha ,\eta_{\alpha },\beta_{\alpha })$      end while


**Figure 5:**
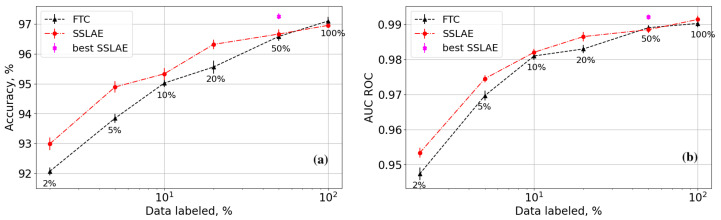
Accuracy (**a**) and AUC ROC (**b**) as functions of the percentage of labeled data for the SSLAE and FTC.

We analyse the accuracy and AUC ROC as functions of the amount of labeled data. For this purpose, we created datasets with 2%, 5%, 10%, 20%, 50%, and 100% labeled data. The results of learning are shown in Figure 5. Each point in the graph represents the result of the parameter optimization using Optuna with 100 trials conducted over 80 epochs. The adjustable parameters included the learning rate, batch size, learning rate reduction parameters for plateaus, and the priority parameter λ. The models were selected based on the validation accuracy, calculated exclusively on labeled samples to avoid data leakage in the SSLAE algorithm. This approach ensured that model selection did not rely on conditionally unlabeled data.

To improve the reliability of the results, we use the bootstrap technique [39] for accuracy and AUC ROC estimation (error bars on the curves in Figure 5). Once the best model was identified based on validation accuracy, we evaluated the bootstrap metrics on the test dataset and calculated the variances of the metrics. Bootstrap helps in obtaining robust statistics by generating repeated samples from the existing dataset. This method involves creating bootstrap samples through sampling with replacement. Each created sample undergoes a metric evaluation, enabling the collection of metric values for subsequent statistical analysis, including confidence interval construction and standard error estimation. We utilized the Poisson strategy as the sampling method for the samples.

From Figure 5a, it can be concluded that there is no significant difference between SSLAE and FTC accuracy when using 50% or more labeled data from the available training dataset. However, with less than 50% labeled data, the SSLAE approach significantly outperforms the FTC approach, with differences exceeding the standard deviation. This improvement is attributed to the autoencoder-based method that uses additional unlabeled data for encoder training. For datasets with 2% and 5% labeled data, the accuracy difference can exceed 1%. The pink dot in the graph represents the best model in the test dataset for SSLAE, which shows lower accuracy in the validation set compared to the red dot.

Figure 5b shows that the AUC ROC curves diverge when less than 50% of the original data are used, favouring SSLAE over FTC. This suggests that SSLAE, leveraging additional unlabeled data, offers an advantage over standard classification with limited labeled data. This improvement stems from the autoencoder’s superior generalization ability and its capacity to represent data in a latent space, making classes more distinguishable. Moreover, the AUC ROC graph indicates that both approaches reach a plateau in this metric as the amount of labeled data increases. Notably, the complexity of the model is high enough to achieve up to 97% accuracy in the test dataset.

**Figure 6:**
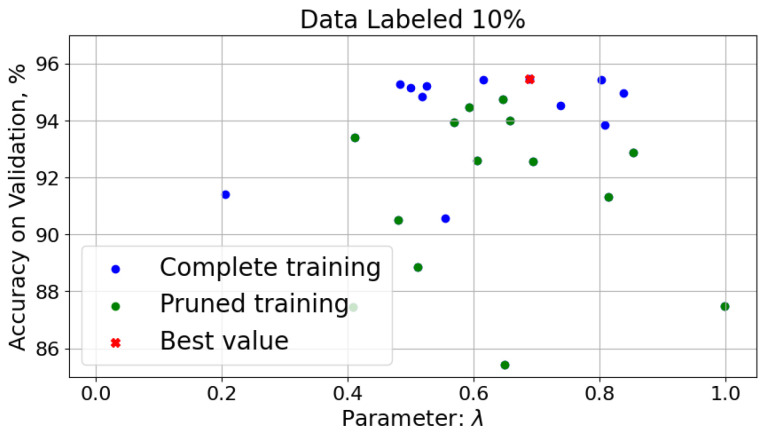
Accuracy as function of the hyperparameter λ for the SSLAE.

The hyperparameter λ governs the balance between the reconstruction loss and the classification loss during training and plays a critical role in guiding the encoder to extract features that are simultaneously informative for reconstruction and discriminative for classification. To analyze the sensitivity of the model to λ, we conducted an additional set of experiments using a dataset with 10% labeled data. In these experiments, we varied λ in the range [0, 1] and recorded the corresponding classification accuracy on the validation set. The results are presented in Figure 6, where the x-axis represents the value of λ and the y-axis shows the classification accuracy on the validation data.

The results indicate a clear dependence of model performance on λ. Specifically, very low values of λ (i.e., prioritizing reconstruction) result in poor classification performance, as the encoder is not sufficiently encouraged to learn discriminative features. Conversely, very high values (approaching 1.0) diminish the benefit of unsupervised learning, especially under low-label regimes. The optimal performance in our case was achieved around λ=0.7, suggesting that a balanced contribution from both loss terms is beneficial for the SSLAE architecture.

## 7. SSLAE Throughput Capacity

In this section, we compare the throughput capacity of the SSLAE approach in inference mode with different benchmark architectures, namely ResNet and EfficientNet. The throughput capacity, which influences the performance and efficiency of a neural network, is crucial for systems like DAS. The speed of obtaining predictions, or model inference, is as important as accuracy, especially for real-time processing of large data sets. A low throughput capacity significantly limits a neural network’s ability to process data in real time, thereby affecting its practical applicability in real-world scenarios.

Throughput capacity is defined as the ability of a neural network to process information, typically measured in operations per second or, in our case, the number of images processed per second. Factors affecting throughput include network architecture, model size, and used hardware. To improve throughput, there are two main approaches. The first involves increasing computational power, which requires significant investment in equipment. The second approach is to reduce the complexity of the model by modifying its architecture, although this may decrease accuracy and generalization ability.

To measure throughput capacity, we first performed a GPU warm-up. This process involved initializing the GPU, warming it up to achieve maximum performance, and caching the necessary data for computations. For an accurate throughput measurement, we applied GPU synchronization, ensuring the completion of all cores in all streams on the CUDA device. Calculations were performed on two different GPUs: ASUS ROG STRIX GTX 1080 Ti GAMING OC, which was introduced in 2017 and has a theoretical power of 12.06 TFLOPS for FP32 and 11 GB of video memory, and GIGABYTE GeForce RTX 4090 WINDFORCE, introduced in 2022, boasts a theoretical power of 82.58 TFLOPS for FP32 and 24 GB of video memory. We focused on GPUs from the mass-market segment, which offers significantly lower costs compared to server-grade GPUs. Conducting computations on a personal computer equipped with mass-market GPUs greatly reduces the technical requirements for perimeter control systems.

**Figure 7:**
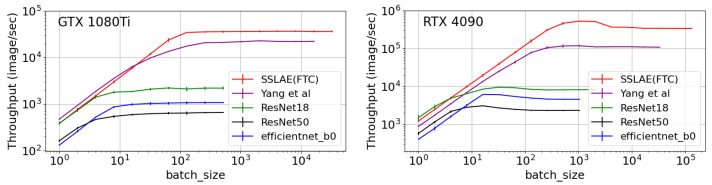
Comparison of throughput capacities for SSLAE and benchmark architectures on the 1080Ti and 4090 GPU. The red curve represents our developed SSLAE model; the purple curve is calculated according to Yang et al. [5]; the green, black and blue curves represent standard models.

Figure 7 displays the comparison of throughput capacities for SSLAE and the benchmark architectures ResNet18, ResNet50, EfficientNet-B0, and Yang et al. [5]. Each data point on the graphs represents the average of 10 experiments, with each experiment measuring the time for 100 batches. From these measurements, the average values and variances were computed. The error bars on the graphs illustrate measurement errors, calculated based on three standard deviations.

The total number of parameters for each architecture is as follows: ResNet18 has 11,689,512 parameters, ResNet50 has 25,557,032 parameters, EfficientNet-B0 has 5,288,548 parameters, and SSLAE(FTC) has 2155 parameters. The input image size for ResNet18, ResNet50, and EfficientNet-B0 is 224×224 pixels with 3 channels, while for SSLAE(FTC), the image size is 96×64 pixels with 3 channels.

The final maximum throughput values, along with their standard deviations, are presented in Table 5. According to the data, the throughput of the SSLAE (FTC) neural network is 36,554 frames per second (FPS) on the GTX 1080Ti graphics card and 526,100 FPS on the RTX 4090. Capturing a single image without overlaps in time and spatial scales takes 4.8 s (recording a line every 0.05 s) from a distance of 102.4 m (sensors spaced 1.6 m apart via optical fibre). Therefore, the number of generated images per second from a single 100 km optical fibre cable is 203. This throughput is significantly more than sufficient for boundary breach prediction, with a delay of approximately 5 s and a spatial resolution of around 100 m.

However, significant losses in boundary breach event detection may occur due to the boundary effects of the neural network in both spatial and temporal windows. In addition, there is a notable delay of up to 5 s between receiving the prediction and the actual boundary breach event. Thanks to the significant throughput of the proposed model, which far exceeds the generation of images without overlaps over a distance of 100 km, it is possible to use a neural network with substantial overlaps. For example, updating event processing every 0.05 s from a sensor every 1.6 m (maximum overlap) results in an image generation rate of 1.25 million FPS from a 100 km optical fibre. When processing images from a 100 km optical fibre with a step of 0.2 s and 6.4 m, a throughput higher than 78,125 FPS is required. Under these conditions, the proposed architecture is sufficient to process data on a GTX 1080Ti graphics card for an optical fibre length of approximately 46 km and for an RTX 4090 graphics card, approximately 673 km.

## 8. Conclusions

In this study, we introduced a hybrid architecture that combines a semi-supervised learned autoencoder with a classifier to enhance the accuracy and efficiency of event classification in infrastructure security systems using distributed acoustic sensors. Our approach effectively leverages both labeled and unlabeled data, allowing the model to learn meaningful representations and improving its generalization capabilities. A distinctive feature of the proposed approach, compared to that in [5], is the principle of training the autoencoder and classifier. Unlike the sequential training method used by the authors in [5], we propose simultaneous training using the SSLAE algorithm. Additionally, we compare the SSLAE and FTC approaches and assess the throughput of the neural network.

Our results demonstrate that the SSLAE model significantly outperforms the conventional fully-trained classifier, especially when the amount of labeled data is limited, showing a notable improvement in leveraging additional unlabeled data. The integrated loss function, which combines elements from both the autoencoder and the classifier, ensures that the encoder extracts features critical for accurate event classification. Analysis of PSNR levels and classification accuracy confirms that the SSLAE approach prioritizes feature extraction relevant to classification, even at the cost of lower reconstruction quality. The SSLAE demonstrates superior throughput capacity compared with benchmark models such as ResNet and EfficientNet, indicating its efficiency in real-time applications.

In general, the proposed SSLAE architecture offers a robust solution for event classification in DAS-based security systems, addressing the challenges of limited labeled data and the need for efficient and accurate real-time monitoring. It is important to note that the classification performance of the proposed SSLAE model may vary across different deployment sites due to varying noise conditions, sensor installation quality, and environmental factors. In our experiments, we observed that test accuracy can range from 92% to over 99%, depending on the specific signal-to-noise ratio and noise profile at each installation. To improve generalization, future work may consider domain adaptation techniques or augmentation strategies that simulate diverse noise environments during training. Additionally, fine-tuning the model with a small amount of labeled data from a new deployment site could further enhance robustness and adaptability in real-world applications.

## Figures and Tables

**Table 2:** Confusion matrix for semi-supervised learned autoencoder. Positive—class “human_step”, PN—Predicted Negative, PP—Predicted Positive.

Class	Train		Valid		Test	
	PN	PP	PN	PP	PN	PP
Negative	239,565	1727	52,574	737	15,365	186
Positive	3897	54,292	1233	10,642	395	3465

**Table 3:** Confusion matrix for classifier with pre-trained encoder: Positive—class “human_step”, PN—Predicted Negative, PP—Predicted Positive.

Class	Train		Valid		Test	
	PN	PP	PN	PP	PN	PP
Negative	229,631	11,611	50,631	2680	14,645	906
Positive	26,802	31,387	5457	6418	1628	2232

**Table 4:** Confusion matrix for fully-trained classifier: Positive—class “human_step”, PN—Predicted Negative, PP—Predicted Positive.

Class	Train		Valid		Test	
	PN	PP	PN	PP	PN	PP
Negative	238,906	2386	52,509	802	15,334	217
Positive	3552	54,637	1047	10,828	353	3507

**Table 5:** Comparison of throughput for SSLAE and benchmark architectures on two different GPUs.

Model	ASUS GTX 1080 Ti		GIGABYTE GeForce RTX 4090	
	Mean, FPS	std, FPS	Mean, FPS	std, FPS
SSLAE (FTC)	36,554	436	526,100	512
Yang et al. [5]	22,814	158	118,346	2281
ResNet18	2220	37	9550	8
ResNet50	661	5	3088	4
EfficientNet-B0	1081	12	6130	54

**Figure A1:**
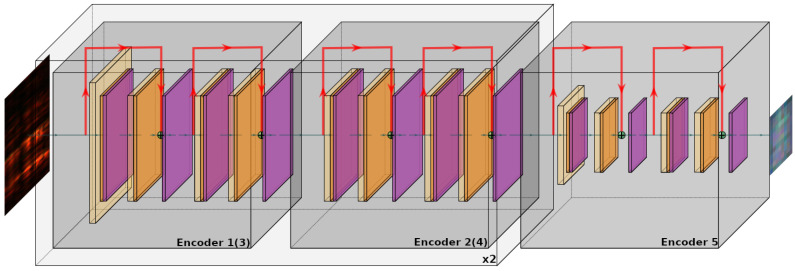
Schematic pipeline of the block “Encoder”: yellow indicates 2D convolution layers; orange—batch normalization layers; purple—ReLU activation function layers.

**Figure A2:**
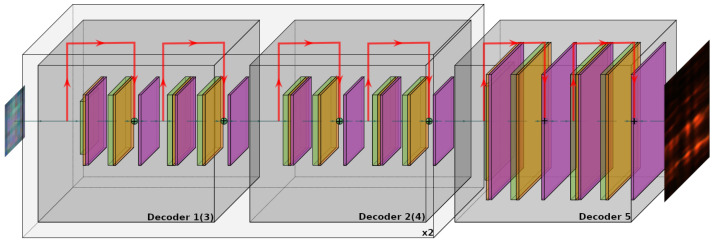
Schematic pipeline of the block “Decoder”: green represents 2D deconvolution layers; orange—batch normalization layers; purple—ReLU activation function layers.

**Figure A3:**
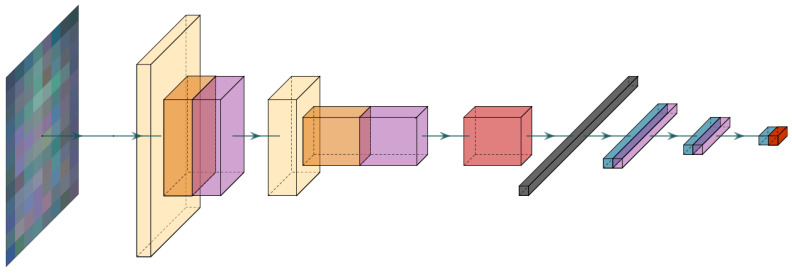
Schematic pipeline of the block “Classifier”: yellow indicates 2D convolution layers; orange—batch normalization layers; purple—ReLU activation function layers; light red—the max-pooling layer; black—the flattening layer; blue—fully connected layers; dark red—the Sigmoid activation function layer.

## Data Availability

No new data were created or analyzed in this study.

## Abbreviations

The following abbreviations are used in this manuscript:

AUC	area under the curve
BCE	binary cross-entropy loss
CNN	convolutional neural network
DAS	distributed acoustic sensors
FC	fully connected layer
FPS	frames per second
FTC	fully-trained classifier
LSTM	long short-term memory
ML	machine learning
MSE	mean squared error loss
PSNR	peak signal-to-noise ratio
ROC	receiver operating characteristic curve
SSAE	sparse stacked autoencoder
SSLAE	semi-supervised learned autoencoder

## Appendix A. Basic Blocks

### Appendix A.1. Block “Encoder”

The encoder is responsible for transforming the raw input data into a lower-dimensional representation while preserving the essential characteristics and extracting the most significant features. Typically, it consists of several convolutional and pooling layers, which help to retain the most relevant information for the classification task. In our work, these compressed data, known as latent representation, are produced by an encoder architecture based on convolutional residual blocks, as shown schematically in Figure A1. The encoder comprises five main sections, each containing two residual blocks with depthwise convolution layers (kernel size 3 × 3, three channels). The first two sections, highlighted in gray, are duplicated sequentially from Encoder 1 to Encoder 4. Encoder 5 is the final block. Table A1 provides a detailed structure of the encoder, including the number of trainable weights. The input to the encoder is an original image of 96 × 64 pixels. A schematic pipeline of the encoder is as follows:

- **Encoder 1**: Reduces the image size to 48 × 32 pixels.
- **Encoder 2**: Reorganizes data without size reduction (48 × 32 pixels, three channels).
- **Encoder 3**: Further reduces the size to 24 × 16 pixels.
- **Encoder 4**: Reorganizes compressed data.
- **Encoder 5**: Compresses data to a final representation size (12 × 8 pixels, three channels).

**Table A1:** Detailed architecture of the block “Encoder”.

Block	Name	Input Shape	Output Shape	Number of Parameters
Encoder 1	Residual Block	96 × 64 × 3	48 × 32 × 3	192
	Residual Block	48 × 32 × 3	48 × 32 × 3	180
Encoder 2	Residual Block	48 × 32 × 3	48 × 32 × 3	180
	Residual Block	48 × 32 × 3	48 × 32 × 3	180
Encoder 3	Residual Block	48 × 32 × 3	24 × 16 × 3	192
	Residual Block	24 × 16 × 3	24 × 16 × 3	180
Encoder 4	Residual Block	24 × 16 × 3	24 × 16 × 3	180
	Residual Block	24 × 16 × 3	24 × 16 × 3	180
Encoder 5	Residual Block	24 × 16 × 3	12 × 8 × 3	192
	Residual Block	12 × 8 × 3	12 × 8 × 3	180

By maintaining a constant number of channels in the latent representation, we significantly reduce the number of trainable parameters. This reduction lowers the complexity of the model, mitigates overfitting, and facilitates training. The total number of trainable parameters, including biases, is 1836, allowing the input image to be compressed by a factor of 64. All models presented in the paper were performed using the PyTorch framework.

### Appendix A.2. Block “Decoder”

The decoder is a key component in the autoencoder architecture responsible for reconstructing the original input from its compressed representation. It typically comprises upsampling and convolutional layers designed to progressively increase the dimensionality of the encoded data, aiming to faithfully reproduce the input data. The architecture of the decoder used in our work is depicted in Figure A2. It consists of five main sections, each containing two residual blocks with deconvolution layers (kernel size 3 × 3, three channels). The first two sections, highlighted in gray, are sequentially duplicated from Decoder 1 to Decoder 4, while the final block is Decoder 5. Table A2 provides a detailed structure of the decoder, including the number of trainable parameters. The input to the decoder is the compressed image output of the encoder, with a size of 12 × 8 pixels.

This decoder architecture mirrors the encoder but utilizes deconvolution layers instead of convolutional layers. In the first, third, and fifth sections, the dimensionality of the image is gradually increased from 12 × 8 pixels back to the original size of 96 × 64 pixels. The total number of trainable parameters in this decoder, including biases, is also 1836.

**Table A2:** Detailed architecture of the block “Decoder”.

Block	Name	Input Shape	Output Shape	Number of Parameters
Decoder 1	Residual Block	12 × 8 × 3	24 × 16 × 3	192
	Residual Block	24 × 16 × 3	24 × 16 × 3	180
Decoder 2	Residual Block	24 × 16 × 3	24 × 16 × 3	180
	Residual Block	24 × 16 × 3	24 × 16 × 3	180
Decoder 3	Residual Block	24 × 16 × 3	48 × 32 × 3	192
	Residual Block	48 × 32 × 3	48 × 32 × 3	180
Decoder 4	Residual Block	48 × 32 × 3	48 × 32 × 3	180
	Residual Block	48 × 32 × 3	48 × 32 × 3	180
Decoder 5	Residual Block	48 × 32 × 3	96 × 64 × 3	192
	Residual Block	96 × 64 × 3	96 × 64 × 3	180

### Appendix A.3. Block “Classifier”

The classifier is responsible for processing the encoded features of the encoder and assigning them to predefined classes. Typically, it consists of fully connected layers followed by activation functions. The classifier is trained using labeled data to distinguish between different types of events detected by the sensor system. In our work, the classifier is specifically tailored for a binary classification task based on the input data received after encoding. It takes the latent representation sized 12 × 8 pixels with three channels as input. The output of this architecture is the pseudo-probability of detecting the positive class. The classifier architecture, depicted in Figure A3, includes both convolutional and fully connected parts.

Table A3 provides a detailed structure of the classifier. The 12 × 8 image with three channels is processed through the convolutional layers (Conv 0, Conv 1) of the classifier. In Conv 0, the image dimensionality is reduced to 6 × 4 pixels, and the number of output channels increases to six. Subsequently, in Conv 1, further dimensionality reduction occurs, with the number of channels increasing to 12. The convolutional part of the classifier concludes with a max-pooling layer (MaxPool2d), resulting in a 1 × 1 image with 12 channels, which is then transformed into a vector of length 12. These vectors are input into fully connected layers (FC 0, FC 1, FC 2) with ReLU or Sigmoid activation functions. Through these layers, the input vectors are sequentially compressed to a size of one, followed by the application of the Sigmoid activation function to obtain the pseudo-probability of detecting the positive class. This function maps values from the range (−∞,+∞) to the interval (0,1). The total number of trainable parameters in the classification part, including biases, is 319.

**Table A3:** Detailed architecture of the block “Classifier”.

Layer	Type	Input Shape	Output Shape	Number of Parameters
Conv 0	Conv2d	12 × 8 × 3	6 × 4 × 6	60
	BatchNorm2d	6 × 4 × 6	6 × 4 × 6	12
	ReLU	6 × 4 × 6	6 × 4 × 6	0
Conv 1	Conv2d	6 × 4 × 6	3 × 2 × 12	120
	BatchNorm2d	3 × 2 × 12	3 × 2 × 12	24
	ReLU	3 × 2 × 12	3 × 2 × 12	0
MaxPool	MaxPool2d	3 × 2 × 12	1 × 1 × 12	0
	Flatten	1 × 1 × 12	12	0
FC 0	Fully Connected	12	6	78
	ReLU	6	6	0
FC 1	Fully Connected	6	3	21
	ReLU	3	3	0
FC 2	Fully Connected	3	1	4
	Sigmoid	1	1	0
